# The impact of immune checkpoint inhibition on atherosclerosis in cancer patients

**DOI:** 10.3389/fimmu.2025.1604989

**Published:** 2025-07-31

**Authors:** Rui Han, Sheng Han Wang, Jingchao Tian, Shanshan Zhou

**Affiliations:** ^1^ Department of Cardiovascular Diseases, First Hospital of Jilin University, Jilin University, Changchun, China; ^2^ Department of Cardiovascular Center, Changchun Vocational College of Health, Changchun, China

**Keywords:** immune checkpoint inhibitors (ICIs), tumor immunity, atherosclerosis, atherosclerotic cardiovascular events (AVEs), immune cells and the signaling pathways

## Abstract

The emergence of immune checkpoint inhibitors (ICIs) have provided a new perspective for cancer immunotherapy. Immune checkpoint inhibitors significantly improve the survival prognosis of patients with various advanced cancers by inhibiting immune checkpoint molecules, thereby releasing the suppression of T cells by tumor microenvironment, such as cytotoxic T-lymphocyte-associated protein 4 (CTLA-4) and programmed cell death protein 1 (PD-1). Immune checkpoint inhibitor (ICI) therapy, while effective, gives rise to distinct immune-related adverse events (irAEs), including cardiovascular toxicities, necessitating focused research efforts to better understand and address these specific complications. The myocarditis-associated toxicity has been extensively studied. This article reviews the latest clinical and preclinical literature on the epidemiology and pathogenesis of ICI-related atherosclerosis, explores the pathophysiological mechanisms by which ICIs promote atherosclerosis, and discusses risk assessment, identification and monitoring methods, and intervention strategies for ICI treatment related atherosclerosis.

## 1 Introduction

Currently, the U.S. Food and Drug Administration (FDA) has approved ICIs such as CTLA-4, PD-1/PD-L1, and lymphocyte-activation gene 3 (LAG-3) for clinical cancer treatment, with many other therapeutic targets under development, such as Cluster of Differentiation 47(CD47), T-cell immunoglobulin and mucin domain-3 (TIM-3), and ITIM domain (TIGIT) (1–3). However, while ICIs activate anti-tumor immunity, they can also disrupt Immune homeostasis of the cardiovascular system (4). Recent studies have found that patients receiving ICI treatment have an increased burden of atherosclerosis. Acute vascular events (AVEs) are increasingly recognized among ICI-treated patients and may significantly impact overall therapeutic benefit and long-term outcomes, although not explicitly been classified as irAEs. Multiple studies have demonstrated that patients who experience adverse vascular events (AVEs) exhibit significantly reduced overall survival. The 30-day mortality rate for arterial events (myocardial infarction or stroke) is significantly higher compared to traditional immune-related adverse events (irAEs) (5–7). Conversely, ICI discontinuation due to AVEs elevates tumor progression risk and causes a median treatment delay. The emerging hypothesis linking immune checkpoint inhibitor therapy to accelerated atherosclerosis and atherosclerotic events necessitates a thorough understanding of how their interactions with the immune system contribute to this pathogenesis. This knowledge will form the foundation for more precise preventive strategies and personalized management of ICI-treated patients, enabling the continuation of effective anticancer therapy without adverse interruptions.

## 2 Clinical studies on ICI treatment and atherosclerotic cardiovascular events

An increasing number of retrospective studies have found that ICIs increase the progression of atherosclerosis and the risk of AVEs. Drobni et al. conducted a large matched cohort study involving 2,842 patients receiving ICI immunotherapy and 2,842 age-matched control patients. The results showed that the risk of AVEs in the ICI treatment group was higher than in the control group not using ICIs (8). Researchers also used PET-CT imaging to find an increased rate of aortic plaque progression, suggesting that ICI treatment may accelerate the progression of atherosclerotic plaques. A registry study by the Oren team on 3,326 patients with solid tumors showed (5) that the cumulative incidence of myocardial infarction and stroke reached 7% 16 months after treatment with the PD-L1 monoclonal antibody atezolizumab. Several short-term ICI treatment retrospective studies also observed an elevated incidence of cardiovascular events. A retrospective study by Bar et al. analyzed the occurrence of AVEs in1,215 non-small cell lung cancer patients receiving ICIs treatment. The results showed the incidence of AVEs within the first 6 months after ICI treatment is higher than that during the 7 to 12 month period (6). The FDA’s pooled analysis of 59 oncology trials showed that, compared to traditional cytotoxic therapy, the incidence of coronary ischemic events within 6 months increased evidently. Since atherosclerosis is a gradually developing chronic inflammatory process that may take years or even decades to manifest clinical symptoms, current data indicate that the risk of atherosclerotic cardiovascular events increases even within a short-term limited follow-up period. Case reports also found similar results. In one case, a patient with metastatic giant cell tumor of the bone experienced a rapid worsening of left circumflex artery stenosis within two months during treatment with the PD-1 monoclonal antibody Pembrolizumab, as observed through dynamic coronary angiography (9). In a 2021 case report, an 83-year-old patient with non-small cell lung cancer experienced acute coronary occlusion 48 hours after starting treatment with the PD-1 monoclonal antibody Pembrolizumab. The patient was diagnosed with non-ST-segment elevation myocardial infarction. Although the patient had previously been diagnosed with severe triple-vessel disease, their clinical condition was stable and asymptomatic (10). Based on existing case studies and retrospective research reports, it can be speculated that ICI treatment may accelerate the progression of chronic plaques and vascular stenosis on one hand, and on the other hand, increase plaque instability, leading to plaque rupture and a higher probability of AVEs (**Table 1**).

**Table 1:** summary table for the clinical studies of ICI therapy and AVEs.

Author	Year	Sample size (n)	Study type	ICI type	Experimental data and main findings
Oren et al. (5)	2020	3326 solid tumor patients (melanoma 21%, lung cancer 19%, kidney cancer 6%)	Single-center retrospective study	PD-1 mAb: atezolizumab	Within 16 months of ICI treatment, incidence of MI was 213 (7%) and stroke was 227 (7%).
BAR et al. (6)	2019	1215 patients (melanoma 40.5%, NSCLC (non-small cell carcinoma)28.7%, urogenital malignancy 10.5%)	Single center retrospective	PD-1 mAbs: pembrolizumab, nivolumab; PD-L1 mAb: atezolizumab; CTLA-4 mAb: ipilimumab	The incidence of AVEs was 2.6% within 6 months after ICI treatment, and the risk during the first 6 months was higher than that in months 7–12 (OR = 3.49, 95% CI: 1.45–8.41; p = 0.002).
Drobni et al. (11)	2020	5684 patients (NSCLC28.8% and melanoma 27.9%as main groups)	Single center retrospective study(matched cohort, case crossover, and imaging study)	Monoclonal antibodies targeting CTLA-4 and PD-1	Study1: Risk of AVEs in ICI-treated group was higher than non-ICI group (HR = 3.3, 95% CI 2.0–5.5; p < 0.001); study2: AVEs increased during 2 years post-ICI vs pre-ICI (HR = 4.8, 95% CI 3.6–6.5; p < 0.001); study3: Annual progression rate of aortic plaques increased from 2.1% pre-ICI to 6.7% post-ICI (p < 0.001), and NCP(non-calcified plaques) annual growth rate reached 11.2%, much higher than 1.6% in control group
Calabretta et al. (12, 13)	2020/2024	20 melanoma patients/47 lung cancer patients	Radiographic cohort study	Monoclonal antibodies targeting CTLA-4, PD-1, and PD-L1	Study1:Aortic target-to-background ratios (TBR)Patients using ICIs significantly increased (TBRpre = 1.76 ± 0.06 vs. TBRpost = 2.05 ± 0.06; P < 0.001). Moreover, significantly enhanced FDG uptake was observed in both non-calcified and mildly calcified atherosclerotic segments of the aorta (P < 0.001). Study2: TBR values of plaque lesions in patients without prior vascular inflammation were significantly higher than those with baseline vascular inflammation (TBRpre = 1.35 ± 0.18 vs. TBRpost = 1.79 ± 0.39; p < 0.001)
FDA-approved pooled analysis of 59 oncology trials (14)	2018	21,664 subjects	Meta-analysis	PD-1 mAbs: pembrolizumab, nivolumab; PD-L1 mAbs: atezolizumab, avelumab, durvalumab	Coronary ischemia risk under ICI treatment increased by 35% compared to traditional cytotoxic chemotherapy (95% CI: 0.76–2.4)

## 3 The role of T cells in the pathophysiology of atherosclerosis

The essence of atherosclerosis is a chronic inflammatory lesion process, where lipoproteins infiltrate the arterial wall through damaged endothelium and undergo modification through oxidation and enzymatic reactions. Simultaneously, the activation of damaged endothelial cells leads to the expression of leukocyte adhesion molecules (such as ICAM-1 and VCAM-1) and the secretion of chemokines, thereby recruiting monocytes to migrate into the vascular wall. Under the regulation of the plaque microenvironment, monocytes differentiate into functionally distinct macrophage subsets. Pro-inflammatory M1 type macrophages are stimulated by oxidized lipids, IFN- *γ*, etc., and secrete IL-1β/IL-6, exacerbating inflammation and leading to lipid phagocytosis, forming foam cells (15). Single-cell RNA sequencing has confirmed that the cellular composition of plaques in mice and humans is nearly identical. In the early stages of plaque progression, macrophages constitute the majority of the plaque’s immune components (16, 17). In unstable plaques prone to rupture, T cell infiltration is significantly increased (18). A 2019 autopsy pathology study involving 11 patients treated with ICI found that, compared to the control group not treated with ICI, the ratio of T lymphocytes to macrophages in atherosclerotic plaques was significantly higher in the ICI-treated group (3). Consistently, the ratio of CD3+ (an immunological marker of T cells) to CD68+ (an immunological marker of macrophages) cells was significantly increased (P=0.002), indicating a shift in atherosclerotic inflammation towards a lymphocyte-dominant type. This autopsy study has limitations due to the small sample size and the inability to completely exclude potential confounding factors. Consistently, Poel et al. observed an increase in CD3+/MAC in the CTLA-4 treatment group in mouse plaques (4), indicating that ICI treatment alters the immune composition within the plaque, shifting the inflammatory response from macrophage-centered to T cell infiltration-dominated, driving the plaque towards a late-stage unstable phenotype.

In atherosclerosis, antigen-presenting cells (APCs) present atherosclerosis-related antigens to naive T cells in lymphoid tissues, leading to the activation of T cells and their migration to plaque areas. Among the T cell subtypes involved in plaque progression, T helper 1 (Th1) cells are the primary CD4+ T cells that promote plaque progression. Th1 cells produce pro-inflammatory cytokines IFN-*γ* and TNF-α, both of which can promote leukocyte recruitment and further production of pro-inflammatory cytokines. Additionally, IFN-*γ* promotes M1 macrophage polarization and foam cell formation (15, 19), TNF-*α* exacerbating endothelial cell damage and oxidative stress. Activated CD8+ T cells induce apoptosis of endothelial cells and smooth muscle within the plaque through the release of perforin and granzymes, triggering endothelial damage and necrotic core expansion (20). In contrast to CD4+ and CD8+ T cells, regulatory T cells (Tregs) can promote the stability of advanced atherosclerotic lesions. Tregs suppress immune responses in atherosclerosis by secreting anti-inflammatory cytokines such as interleukin-10 (IL-10) and TGF-β, and they can also maintain the integrity of the fibrous cap by inhibiting Th1 cell activity, reducing macrophage activation, and preventing collagen degradation (21). The role of activated Th17 cells in atherosclerosis remains controversial (22, 23). Clinical studies have shown that the cytokine IL-17 secreted by Th17 cells can synergize with IFN-γ to increase IL-6 secretion and promote inflammation. An imbalance in the Th17/Treg cell ratio (increased Th17 and decreased Treg) has been shown to be significantly associated with the progression of atherosclerosis.

## 4 Preclinical studies on ICIs promoting atherosclerosis

### 4.1 Immune checkpoint inhibition targeting T cells

Immune checkpoints are co-stimulatory or co-inhibitory molecules primarily expressed on the surface of immune cells such as T cells and antigen-presenting cells (APCs). They transmit "brake" signals through receptor-ligand interactions, limiting the overactivation of immune responses. Immune checkpoints such as CTLA-4, PD-1/PD-L1, and lymphocyte activation gene 3 (LAG-3) can be expressed on the surface of T cells, blocking the activation signals of T cells and preventing their activation (24). In cancer treatment, immune checkpoint inhibitors (ICIs) target and inhibit immune checkpoints, lifting their suppression on T cell activation (1), promoting systemic T cell-mediated anti-tumor responses, but also leading to immune off-target events. It is currently believed that ICI treatment activates T cells that recognize atherosclerosis-specific autoantigens, leading to the clonal expansion of autoreactive CD4+, CD8+, and other T cell subsets (11). The activated T cells accelerate the chronic progression of plaques and increases their instability through interaction with smooth muscle cells and macrophages in the plaque microenvironment (25). The clonal expansion of T cells may result from the reactivation of exhausted T cells and the recruitment of newly activated T cells by ICI treatment. In both atherosclerotic plaques and cancer, T cells that have undergone functional exhaustion under prolonged antigen stimulation, characterized by high PD-1 expression, have severely impaired immune function (26, 27). Studies have shown that ICI treatment can expand and differentiate stem-like precursor exhausted T cells (TCF1+PD-1+ cells) within the exhausted T cell population into functional effector T cells, restoring immune responses (28, 29). However, there are certain limitations to the reactivation of these exhausted T cells. Evidence has shown that PD-1 inhibition can upregulate chemokines such as CXCL9/10, recruiting the migration of newly activated effector T cells through CXCR3 signaling (30). Further research is needed to determine the roles of T cell recruitment and T cell reactivation in ICI-related atherosclerosis.

#### 4.1.1 PD-1 and CTLA-4

**Figure 1:**
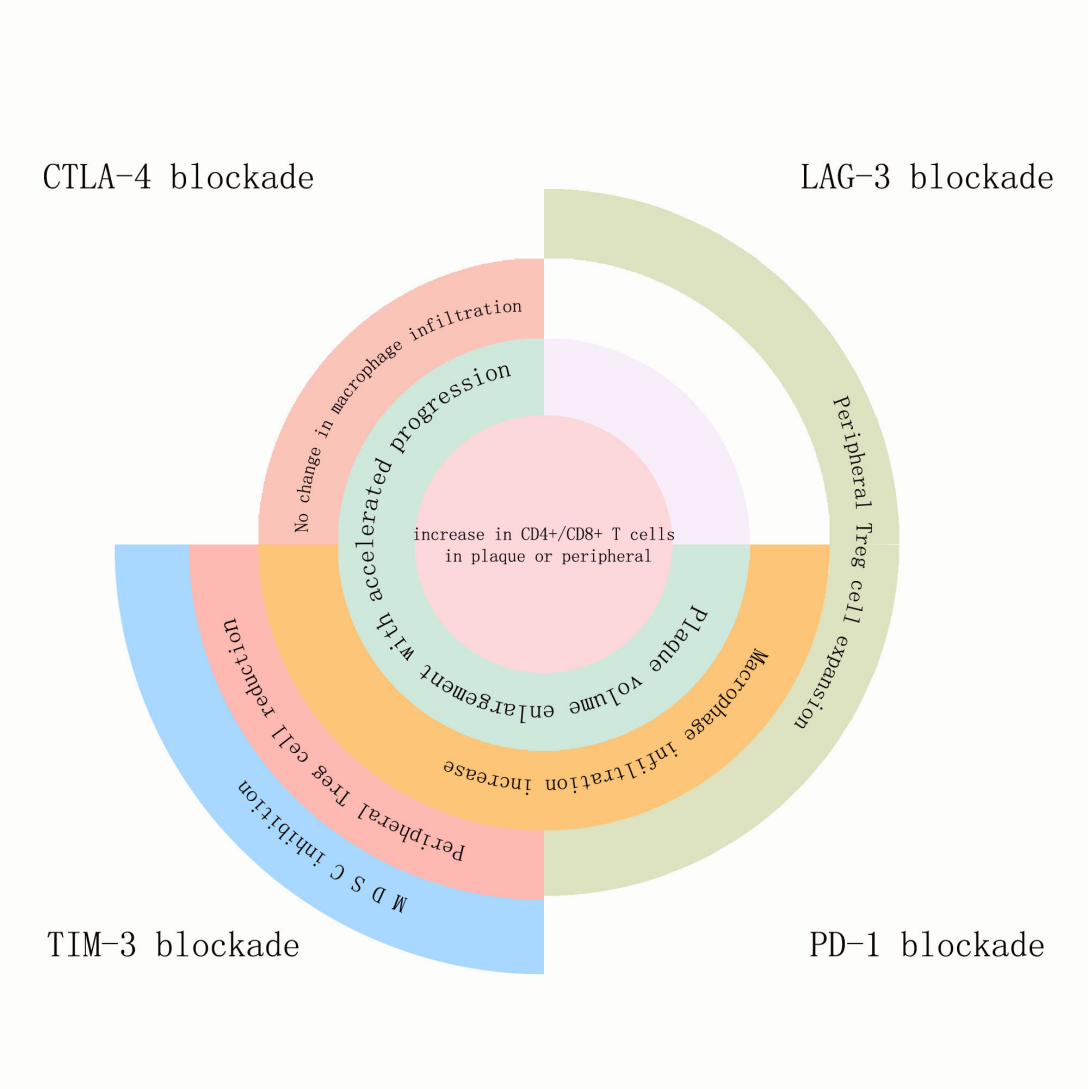
The effects of PD-1, LAG-3, TIM-3, and TIGIT blockade on atherosclerotic plaques and the immune components within the plaques, as well as on immune cells inside or outside the plaques: Blockade of all four immune checkpoints promoted the expansion of CD4+/CD8+ T cells either within the plaques or in peripheral circulation. Blockade of CTLA-4, TIM-3, and PD-1 accelerated plaque volume growth and progression. Blockade of CTLA-4 did not affect macrophage infiltration, while blockade of TIM-3 and PD-1 increased macrophage infiltration within the plaques. TIM-3 blockade reduced peripheral Treg cells, whereas blockade of LAG-3 and PD-1 pathways increased peripheral Treg cells. TIM-3 blockade uniquely suppressed MDSCs (myeloid-derived suppressor cells).

PD-1 is primarily expressed on T cells as a member of the B7 family on T cells. It can bind to programmed death ligand 1 (PD-L1) and programmed death ligand 2 (PD-L2) expressed on APCs (31). The PD-1 pathway mainly exerts a negative regulatory effect during the effector phase of T cell activation (32). The binding of PD-1 to PD-L1/PD-L2 recruits Src homology phosphatases (SHP-1/SHP-2) to counteract the T cell activation signals triggered by the T cell receptor (TCR) and CD28 on the T cell surface (33). Current research indicates that upregulation of PD-1 expression can reduce plaque volume and inhibit T cell activation. The interaction between PD-1 and PD-L1 can suppress the activation of effector CD4+ and CD8+ T cells, while promoting the differentiation of naive CD4+ T cells into FoxP3+ Treg cells and maintaining the activity of Treg cells (34, 35). In human atherosclerotic plaques, T cells expressing PD-1 are mainly concentrated in the shoulder region of the necrotic core, and the percentage of PD-1 expression in plaques is positively correlated with the immune cell infiltration score. The level of PD-1 high-expressing T cells is significantly higher in unstable plaques than in stable plaques (36). Clinical data show that the overall expression levels of PD-1 and PD-L1 are reduced in patients with coronary artery disease (CAD) and acute coronary syndrome, further confirming the protective role of the PD-1 pathway in the development and progression of atherosclerosis (37). Studies on genetic knockout models indicate that defects in the PD-1/PDL1/PDL2 genes exacerbate atherosclerosis in hyperlipidemic Ldlr-/- mice and induce an inflammatory plaque phenotype characterized by increased CD4+ and CD8+ T cells and macrophages. At the cellular level, macrophages and DCs from PD-L1-deficient Ldlr-/- mice are more likely to activate T cells after antigen presentation compared to those from control mice, and CD8+ T cells from PD-1-deficient Ldlr-/- mice are more cytotoxic. Notably, Cochain et al. found a significant increase in Treg cells in PD-L1/PD-L2-deficient mouse models, yet the plaques still showed significant progression, indicating that the inhibitory effect of the PD-1 pathway on activated CD4+/CD8+ T cells outweighs the protective effect of Treg cells on atherosclerosis (38–40) (**Table 2**) (**Figure 1**).

**Table 2:** Summary of mouse models of atherosclerosis induced by PD-1 and CTLA-4 pathway inhibition.

Research	Model	The role in atherosclerosis	Impact on immune cells
Bu et al. Gotsman et al. (38, 39)	Ldlr-/- mouse model with PD1/PD-L1/PD-L2 gene defects	Atherosclerotic plaque volume increases, progression accelerates	Increased infiltration of CD4+ and CD8 T cells and macrophages in plaque lesions, with higher expression of IFN-g and TNF-a
Cochain et al. (40)	Ldlr-/- mouse model with PD-L1/PD-L2 gene defects	Atherosclerotic plaque volume increases, progression accelerates	Systemic CD4^+^ and CD8^+^ T cell and Foxp 3^+^ *T*_reqs_ cell expansion, massive T cell infiltration in plaque lesions
Karin van Dijk et al. (36)	Apoe-/- Leiden mouse model undergoing venous bypass surgery treated with PD-1 monoclonal antibody	No significant changes were detected.	An elevation in TRM (tissue-resident memory T cell) infiltration within venous grafts demonstrated a concordant increase with the progression of vascular inflammation.
Kitano et al. (41)	Apoe-/- mouse model with CTLA-4 overexpression	Reduction in atherosclerotic lesions (without affecting smooth muscle and collagen content in plaques)	Decreased number of CD4+ T cells and macrophages in plaques, and reduced systemic T cell activation levels
Poels et al. (42)	Ldlr-/- mouse model treated with anti-CTLA-4 antibody	Atherosclerotic plaque volume increases, progression accelerates, plaques shift to late unstable phenotype, necrotic core expands	Increased CD+3 T cells in plaques, elevated CD3+/MAC ratio, increased CD4+ T cells in spleen and circulation

The inhibition of the PD-1 signaling pathway is also closely related to the abnormal activation of tissue-resident memory T cells (TRM) in atherosclerotic plaques (36). TRM cells have the characteristics of long-term tissue residence and rapid response to antigen stimulation, and they highly express various inhibitory checkpoint molecules (such as PD-1, CTLA-4, and LAG-3), playing an important role in anti-tumor immunity such as melanoma. After receiving ICI treatment, TRM cells residing in the tumor are reactivated and expanded, releasing perforin and granzyme to directly lyse tumor cells; on the other hand, in the atherosclerotic microenvironment, activated TRM cells secrete pro-inflammatory factors TNF- α and IFN- γ, which amplify chronic inflammatory responses and directly damage the fibrous cap structure of the plaque by perforin and granzyme, thereby exacerbating plaque instability. K. Van Dijk et al. found that hypercholesterolemic Apoe-/- mice that underwent venous bypass surgery and received PD-1 monoclonal antibody treatment showed a significant increase in vascular inflammation and a corresponding increase in TRM cell infiltration in the venous grafts, indicating that TRM cells are involved in the exacerbation of ICI-related atherosclerotic inflammation.

A recent animal study has shown that PD-1 inhibition therapy exacerbates cardiac injury during the ischemia-reperfusion injury phase of myocardial infarction (43). Hess et al. used C57BL/6J mice to construct a reperfusion acute myocardial infarction (repAMI) model by ligating the left coronary artery followed by reperfusion. Mice in the anti-PD-1 treatment group received regular injections of PD-1 antibodies before repAMI induction, while the control group was treated with IgG2a. The results showed that anti-PD-1 treatment before reperfusion significantly increased the infiltration of CD8+ T cells in the myocardial tissue of mice (an increase of 33.6%). This suggests that inhibiting the PD-1/PD-L1 pathway exacerbates injury during the reperfusion phase of myocardial infarction. Interestingly, Z. Varga’s team confirmed that reversible myocardial ischemic injury also aggravates the cardiotoxic effects induced by PD-1 inhibition (44). Researchers induced reversible cardiac ischemia in C57BL/6J mice using isoproterenol (ISOP) and administered PD-1 inhibition therapy after their recovery period. The results showed that compared to mice without ischemic injury, mice in the reversible cardiac ischemia group had significantly increased infiltration of T cells and macrophages in the myocardium after anti-PD-1 treatment, along with upregulated expression of pro-inflammatory cytokines (such as IL-17A and IFN- *γ* ). This indicates that reversible myocardial ischemia exacerbates the cardiotoxicity and cardiovascular inflammatory response triggered by anti-PD-1 antibodies. However, its impact on the progression of atherosclerotic plaques requires further study.

Similar to PD-1 and PD-L1, cytotoxic T lymphocyte-associated protein 4 (CTLA-4) is also an important negative regulator of T cell activation. Unlike PD-1, CTLA-4 is primarily expressed on Treg cells (45) and exerts its inhibitory effect by blocking T cell receptor ( TCR ) signaling during the early stages of T cell activation. When the T cell receptor (TCR) and CD28 on the surface of T cells interact with the MHC-peptide complex and CD80/CD86 on antigen-presenting cells, CTLA-4 binds to CD80/CD86 with higher affinity than CD28, thereby competitively inhibiting the co-stimulatory signals mediated by CD28 and reducing T cell activity. Additionally, CTLA-4 expressed on Treg cells can mediate the "trans-endocytosis" of Treg cells, removing CD80/CD86 molecules from antigen-presenting cells and rendering the co-stimulatory signals mediated by CD28 ineffective (45).

**Figure 2:**
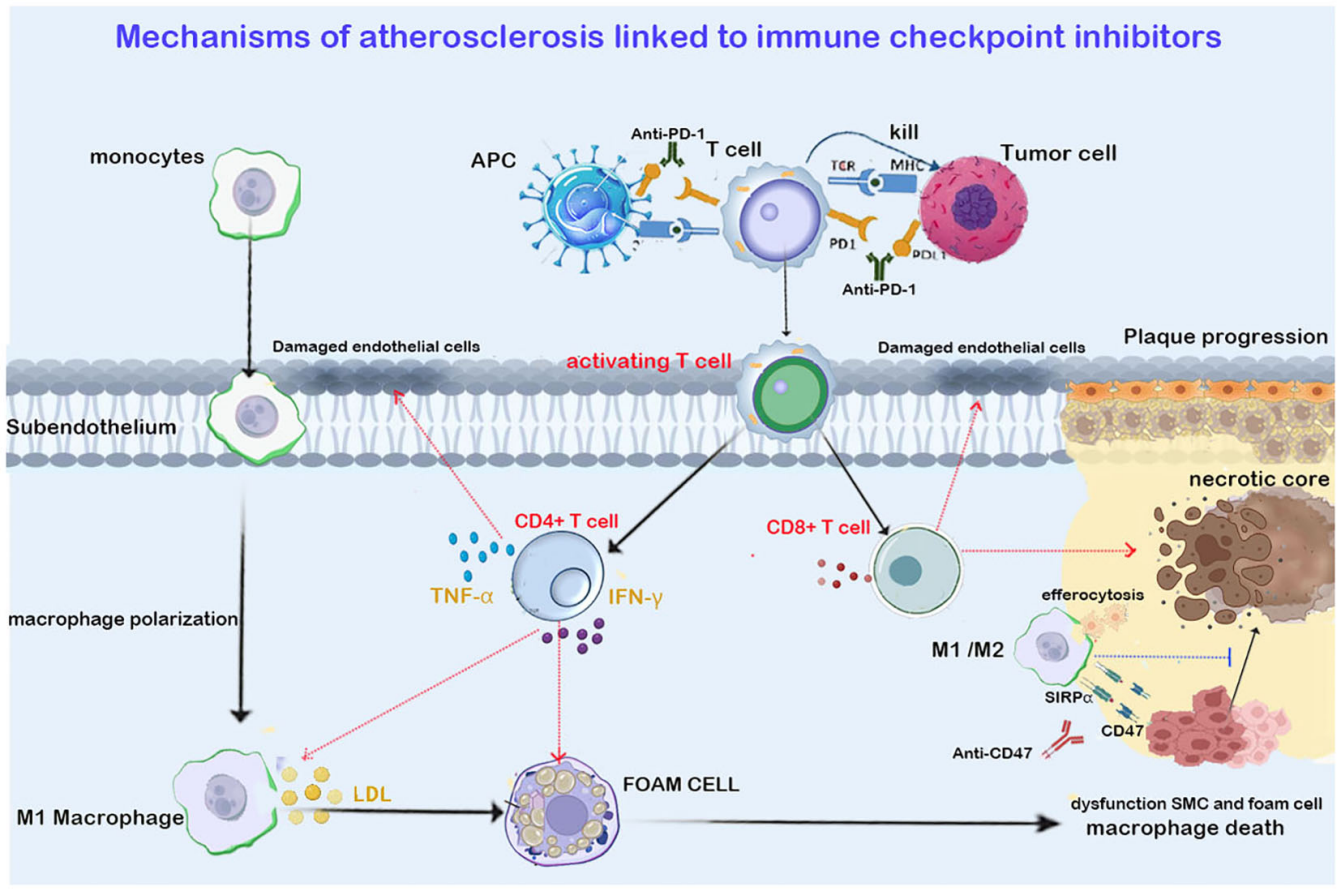
Shows that ICIs targeting T cells (anti-PD1/PDL1 in the figure) promote the infiltration of CT4+ Th1 and CD8+ cells into the plaque. Th1 cells exacerbate plaque progression by secreting IFN-*γ* and TNF- *α*. IFN-*γ* promotes M1 macrophage polarization and foam cell formation. TNF- *α* aggravates arterial endothelial cell damage and oxidative stress. CD8+ T cells trigger endothelial injury and necrotic core expansion by releasing perforin and granzymes. The immunosuppressant targeting macrophage efferocytosis (anti-CD47 in the figure) upregulates the phagocytic function of macrophages, clearing apoptotic vascular smooth muscle cells (VSMCs) and foam cells, thereby inhibiting necrotic core expansion and reducing the plaque. Red arrows in the figure indicate promotion, while blue arrows indicate inhibition.

T cell-specific CTLA-4 overexpression significantly reduces atherosclerotic lesions in Apoe-/- mice, limits the number of CD4+ T cells and macrophages in plaques, and decreases systemic T cell activation levels (41) (**Table 2**). In contrast, in the Ldlr-/- mouse model receiving CTLA-4 inhibitory therapy, the burden of atherosclerotic plaques significantly increased (plaque area in the aortic region increased by 2.0 times), and the plaques exhibited characteristics of high instability and rupture risk, including reduced collagen and smooth muscle cell content, intimal thickening, and expansion of the necrotic core area (42, 46). The number of CD+3 cells/MAC increased in mouse plaques, and flow cytometry showed no significant change in the number of monocytes in the spleen tissue of mice. Consistently, 18F-fluorodeoxyglucose (FDG) PET/CT imaging did not show increased inflammation in the aorta, spleen, or bone marrow, suggesting that CTLA-4 inhibition does not affect monocyte and macrophage-driven vascular inflammation. The number of CD4+ T cells in the mouse spleen increased, while the number of CD8+ T cells and regulatory T cells remained unchanged. This indicates that CTLA-4 inhibition, like PD-1 inhibition, induces an activated phenotype of T cells, and the inhibition of different immune checkpoint pathways has differential effects on the activation of immune cell components in atherosclerosis (**Figure 2**).

Notably, the latest study by Jan Nilsson et al. revealed a new mechanism by which CTLA-4 inhibition mediates cardiotoxicity. Researchers injected CTLA-4 antibodies into mice experiencing heart failure induced by transverse aortic constriction (TAC), confirming that CTLA-4 inhibition promotes CXCR4-mediated Th17 cell differentiation and activation, upregulating IL-17A production (47). The pro-atherogenic role of IL-17A has been confirmed by multiple experiments (48), and future studies are needed to further prove whether CTLA-4 inhibition can influence the progression of atherosclerosis through the CXCR4/Th17/IL17A axis.

#### 4.1.2 The new generation of immune checkpoints: LAG-3, TIM-3, and TIGIT

LAG-3, TIM-3, and TIGIT are co-inhibitory molecules expressed in T cells, NK cells, macrophages, and dendritic cells, representing the new generation of ICI therapeutic targets following CTLA-4 and PD-1 (49, 50). In the tumor microenvironment (TME), exhausted CD8+ T cells (Tex) simultaneously overexpress PD-1, TIM-3, LAG-3, and TIGIT. These co-inhibitory molecules synergistically promote tumor immune evasion. Blocking TIM-3, LAG-3, and TIGIT can synergize with PD-1 blockade to restore the proliferative capacity of exhausted CD8+ T cells in tumors and upregulate NK cell activation, thereby enhancing tumor-killing effects (49–52). Currently, combination therapies targeting these three molecules with PD-1 have been extensively studied in cancer treatment. The combination of PD-1 and LAG-3 blockade has been approved for patients with unresectable or metastatic melanoma. PD-1 and LAG-3 double knockout CD8+ T cells exhibit higher TCR diversity, stronger cytotoxicity (increased expression of GZMB and PRF1), and IFN-*γ* dependent anti-tumor effects (53, 54), while the combination of PD-1 with TIM-3 or TIGIT blockade is still in the exploratory stage. Currently, PD-1/TIM3 and PD-1/TIGIT bispecific antibodies have entered clinical trials and have shown better therapeutic effects than monotherapy, requiring more preclinical and clinical research support (55, 56). However, the inhibition of LAG-3, TIM-3, and TIGIT also carries the risk of promoting the development of atherosclerosis (**Figure 1**).

##### 4.1.2.1 LAG-3

Lymphocyte Activation Gene 3 (LAG-3), as a structural homolog of CD4, is induced and maintained in expression upon T cell stimulation. LAG-3 inhibits T cell activation by binding to MHC class II molecules and the ligand Galectin-3 (49). A cohort observational study showed elevated LAG-3 levels in patients with coronary heart disease, indicating that LAG-3 is a potential predictor of coronary heart disease risk (57). In the Ldlr-/- hyperlipidemia mouse model, whether it was LAG-3 deletion, LAG-3 monotherapy blockade, or LAG3/PD-1 dual-target inhibition, although it did not increase the plaque burden in Ldlr-/- mice, it increased the accumulation of CD4+ T cells in arterial plaques and the vascular adventitia, and expanded the populations of CD4+ and CD8+ effector T cells and Tregs in the spleen and peripheral blood circulation, along with increased IFN-y production. Additionally, LAG3/PD-1 dual-target inhibition showed significant synergistic effects in this regard (58).

##### 4.1.2.2 TIM-3 and TIGIT

TIM-3 and TIGIT are cutting-edge targets for ICI therapy. TIM-3 is primarily expressed on Th1 cells and CD8+ T cells, inhibiting T cell activity by binding to ligands such as Galectin-9, regulating the differentiation of monocytes into macrophages and the function of natural killer (NK) cells (49). The expression of LAG-3 is upregulated in atherosclerotic lesions (59). Foks et al. demonstrated that Anti-TIM-3 treatment primarily drives the activation of macrophages and T cells, promoting the progression of atherosclerosis in Ldlr-/- mice. Compared to the control group, Anti-TIM-3 treatment increased the development of atherosclerosis in the aortic root by 35% and in the aortic arch by 50% in Ldlr-/- mice (60). *In vitro* experiments confirmed that mouse macrophages exposed to TIM-3 antibodies and loaded with oxidized low-density lipoprotein (oxLDL) significantly enhanced the secretion of monocyte chemoattractant protein-1 (MCP-1). Compared to the control group, Anti-TIM-3 treatment increased the expression of MCP-1 in atherosclerotic lesions by approximately 2-fold, leading to increased infiltration of circulating monocytes and macrophages in the atherosclerotic plaques of Ldlr-/- mice treated with Anti-TIM-3. Anti-TIM-3 antibody treatment also resulted in decreased levels of regulatory T cells (Tregs) in the spleen, peripheral blood, and atherosclerotic lesions of Ldlr-/- mice, and increased the total number and activated percentage of peripheral CD4^+^T cells, producing more interleukin-17 (IL-17) (60) (**Table 3**).

**Table 3:** Summary of mouse models of atherosclerosis induced by LAG-3, TIM-3, and CD47 pathway inhibition.

Research	Model	The role in atherosclerosis	Impact on immune cells
Engelbertsen et al. (58)	Ldlr-/- mouse models treated with LAG-3 antibody and LAG-3 gene knockout Ldlr-/- mouse models	No increase in plaque burden was observed in Ldlr-/- mice	Accumulation of CD4+ T cells in arterial plaques and vascular adventitia, expansion of CD4+ and CD8+ effector T cells and Tregs in the spleen and peripheral blood circulation of mice, and increased plasma IFN-y
Yagita et al. (60)	Ldlr-/- mouse models treated with Tim-3 antibody	Increased volume and accelerated progression of atherosclerotic plaques, no significant change in the content of apoptotic or necrotic cores in plaque lesions	Increased number of circulating monocytes and macrophages in atherosclerotic plaque lesions, decreased Tregs levels in the spleen, blood, and atherosclerotic lesions of mice, increased total number and activated percentage of peripheral CD4^+^ T cells, and increased production of interleukin-17 (IL-17)
Kojima et al. (61)	Apoe-/- mouse models treated with CD47 antibody	Plaque area in the aortic sinus and aorta of mice reduced, and significantly reduced the number of apoptotic cells within the plaques	Enhanced efferocytosis of macrophages (*in vitro* experiments)
Singla et al. (62)	Mouse models with deletion of signal regulatory protein α (Sirpa)	Regression of plaques and reduction of necrotic core areas were observed in the atherosclerotic plaques of mice	Enhanced efferocytosis of macrophages (*in vitro* experiments)

Additionally, it is noteworthy that TIM-3 can be expressed on myeloid-derived suppressor cells (MDSCs). Under pathological conditions such as tumors or chronic inflammation, chronic inflammatory signals (e.g., cytokines IL-6, GM-CSF) can lead to blocked differentiation of myeloid cells, resulting in the massive expansion of immature cells that enter the peripheral circulation, forming MDSCs with immunosuppressive functions. These MDSCs can block T cell function by secreting immunosuppressive cytokines (e.g., TGF-β and IL-10), leading to reduced T cell function. In non-small cell lung cancer models, it has been confirmed that inhibiting TIM-3 can reduce the infiltration of MDSCs in the tumor microenvironment, upregulate T cell activity, and enhance tumor immune response (60, 63). In atherosclerosis research, by transferring CD11b+ Gr-1+ (myeloid markers of MDSCs) cells into Ldlr-/- mice fed a Western diet, it was confirmed that MDSCs have a protective role in atherosclerosis. The results showed that this treatment reduced atherosclerotic plaque formation in the aortic root by 35% and decreased the number of Th1 and Th17 cells in the spleen by 50% (64, 65). However, whether TIM-3 blockade reduces MDSC infiltration in the chronic inflammatory environment of plaques and exacerbates plaque progression still requires further experimental validation.

T cell immunoreceptor with Ig and ITIM domains (TIGIT), also known as Vsig9/Vstm3/WUCAM, is a novel co-inhibitory molecule. TIGIT is transiently expressed after T cell receptor (TCR) stimulation and stably exists on regulatory T cells (Tregs) and dysfunctional CD8+ T cell subsets. By competing with the co-stimulatory molecule CD226 for binding to the ligand CD155, TIGIT inhibits the CD226-mediated T cell activation signal, thereby weakening T cell cytokine secretion and proliferation capacity (66, 67), in addition to its combination with PD-1 inhibitors in anti-tumor therapy, recent studies have found that combined blockade of CD47 and TIGIT targets can enhance the phagocytic activity of macrophages against leukemia *in vitro*. TIGIT blockade can stimulate phagocytosis by repolarizing M2-type macrophages to M1-type macrophages, synergizing with CD47 antibodies that block the "don’t eat me" signal, inducing macrophage phagocytosis of acute myeloid leukemia (AML) cells. This has been validated in allogeneic experiments using AML cell lines and autologous primary monocyte experiments from AML patients (68).

Currently, there is no mouse model study on the effects of TIGIT blockade on arterial plaque burden and changes in immune cell components within it. However, it has been confirmed that TIGIT+Tregs can secrete sFGL2 to inhibit pro-inflammatory Th1/Th17 responses, promote anti-inflammatory Th2 responses, and upregulate the secretion of anti-inflammatory factors IL-10 and TGF-β. The TIGIT-CD155 pathway induces the transition of M1 macrophages to M2 macrophages, reducing plaque inflammation and plaque instability (69–71).This suggests that TIGIT blockade may impair the immunosuppressive function of Tregs, upregulate M1/M2, accelerate lipid deposition, and increase the risk of plaque instability and rupture. Xinlin Xiong et al. first revealed through flow cytometry that TIGIT+ regulatory T cells (TIGIT+Tregs) in patients with acute coronary syndrome (ACS) are significantly reduced (significantly lower than in patients with chronic coronary syndrome (CCS) and healthy controls, P<0.05). Multivariate logistic regression analysis has confirmed that TIGIT+Tregs are an independent predictor of ACS (OR = 0.902, P=0.001) (72).

#### 4.1.3 Emerging therapeutic target CD300Id

PMN-MDSCs (polymorphonuclear myeloid-derived suppressor cells) are a subtype of MDSCs with stronger T cell inhibitory capabilities (73) than other MDSC subtypes. In the latest cancer therapy research, CD300ld is specifically highly expressed on PMN-MDSCs and regulates the recruitment and immunosuppressive function of PMN-MDSCs through the downstream STAT3-S100A8/A9 signaling axis. Blocking CD300ld significantly reduces STAT3 phosphorylation levels, decreases S100A8/A9 transcriptional activation, thereby inhibiting the infiltration of PMN-MDSCs into the tumor microenvironment. Blocking CD300ld can significantly increases the number of effector immune cells such as CD8+ T cells, CD4+ T cells, and natural killer cells, reversing the immunosuppressive state of the tumor. This demonstrates potential in tumor therapy. Current studies indicate that the number of PMN-MDSCs is significantly reduced in the late stages of atherosclerosis, and their reduction may weaken the inhibition of atherosclerotic inflammation, promoting plaque development. Whether CD300Id blockade promotes atherosclerosis by reducing PMN-MDSC infiltration remains to be further explored experimentally (64, 73).

### 4.2 Immune checkpoint inhibition targeting efferocytosis

#### 4.2.1 CD47

The CD47-SIRPα signaling pathway is a unique immune checkpoint, different from PD-1 and CTLA-4. CD47 is widely expressed on cell membranes. By binding to signal regulatory protein *α* (SIRP *α* ) on the surface of phagocytes such as macrophages and dendritic cells (DCs), it inhibits the phagocytic function of these cells. CD47 is rapidly downregulated during apoptosis, allowing phagocytes to perform efferocytosis and clear apoptotic cells through programmed cell removal. Cancer cells overexpress CD47 to block this process (74). Anti-CD47 antibodies can block the CD47-SIRPα pathway, restoring the phagocytic function of macrophages and enhancing their ability to recognize and kill tumor cells (75, 76). They also promote timely efferocytosis to clear apoptotic cells, reduce the release of inflammatory factors, and prevent chronic inflammation from promoting tumor progression. Currently, the humanized CD47 monoclonal antibody (magrolimab) has shown significant tumor volume reduction in patients with relapsed/refractory lymphoma and has received FDA breakthrough therapy designation (45, 74).

In atherosclerotic arteries, defective efferocytosis leads to the expansion of the necrotic core of plaques. The expression of SIRP *α* and CD47 is increased in human atherosclerosis. Signal regulatory protein *α* is primarily localized in macrophages within atherosclerotic arteries, while CD47 is strongly localized in the necrotic core of plaques (61, 62).CD47 Inhibition therapy reduces atherosclerosis by restoring efferocytosis in plaques, removing apoptotic vascular smooth muscle and foam cells (61, 77) (**Figure 2**). In the Apoe-/- mouse model of apolipoprotein E deficiency by Komoji et al., CD47 antibody treatment reduced plaque area in the aortic sinus and aorta and significantly decreased the number of apoptotic cells within the necrotic core (61) (**Table 3**). Similar results were obtained in mouse model experiments by Paul et al. Paul et al. also found that the pro-efferocytosis receptor low-density lipoprotein receptor-related protein 1 (LRP1) in macrophages is necessary for the enhancement of efferocytosis by anti-CD47 antibodies, limiting the formation of atherosclerosis and reducing the formation of the necrotic core (78).

Specific knockout experiments provide new insights into the mechanisms by which CD47 inhibition therapy affects atherosclerosis. In experiments with endothelial-specific CD47 knockout (CD47iECKO) mice, the plaque area in the aortic sinus of CD47iECKO mice was significantly reduced, and single-cell sequencing results showed increased macrophage infiltration in the plaques. *In vitro* experiments confirmed that the loss of endothelial CD47 enhances the phagocytic ability of endothelial cells towards apoptotic Jurkat cells, while upregulating the expression of phagocytosis-related receptor genes such as FasL and MerTK (79). Singla et al. unexpectedly found that myeloid cell-specific CD47 knockout Apoe^-/-^ mice exhibited exacerbated atherosclerosis. Contrary to previous experimental views, Singla et al. suggest that CD47 signaling in myeloid cells such as macrophages may have a protective role against atherosclerosis *in vivo*, and that systemic CD47 inhibition may reduce atherosclerosis by suppressing smooth muscle cell CD47 expression and stimulating efferocytosis. To date, no experiments have investigated the role of smooth muscle cell CD47 in atherosclerosis, which remains a subject for future research. Additionally, Singla and Flores et al. confirmed that the loss of SIRP *α* signaling can also reduce atherosclerotic plaques (77). Plaque regression and a reduction in the necrotic core area were observed in both systemic SIRPα-deficient Apoe^-/-^ mice and myeloid cell-specific SIRPα-deficient Apoe^-/-^ mice. Current anti-CD47 therapy has been shown to cause side effects including erythrocytopenia, hemoglobin reduction, and thrombocytopenia by blocking the binding of CD47 to TSP-1 (62). Hematological analysis by Singla et al. revealed that compared to wild-type and signal regulatory protein *α* knockout mice, CD47 knockout mice had reduced red blood cell counts and hemoglobin levels, suggesting that selectively blocking signal regulatory protein *α* (SIRP *α* )-mediated signaling could circumvent the hematological side effects of using CD47-Ab, making it an effective ICI treatment strategy for cancer patients at high risk of atherosclerosis.

It is noteworthy that the enhancement of efferocytosis by myeloid phagocytes such as macrophages affects T cell function (45). Cytokines such as TGF-*β* released by myeloid cells during the phagocytosis of apoptotic debris can induce the clonal expansion of Tregs, promoting immune tolerance. Meanwhile, lactate, TGF-β1, and tryptophan metabolites produced by efferocytosis can collectively upregulate the expression of PD-1 on Tregs and CD8+ T cells. This suggests that CD47 inhibition therapy-enhanced efferocytosis may promote cancer immunosuppression by expanding Tregs and enhancing PD-1 expression on T cells, thereby weakening anti-tumor effects. Several studies have shown that the combination of CD47 monoclonal antibody (Magrolimab) and PD-1 antibody can significantly reduce tumor size (80, 81). Future research is needed to optimize the therapeutic benefits of CD47 inhibition therapy in both cancer and atherosclerosis.

Currently, various novel CD47 antibody therapies have entered clinical trials. The CD47 inhibitor BRB-002 has shown dose-dependent anti-AS effects in Apoe-/- mouse models, holding promise for achieving dual therapeutic effects in both cancer and atherosclerosis in further clinical trials (82). At the molecular mechanism level, recent studies have found that microRNA-299-3p is significantly downregulated in patients with atherosclerosis and coronary heart disease. Target gene prediction has confirmed that microRNA-299-3p can specifically recognize and bind to the "CCCACAU" conserved sequence in the 3’UTR region of human CD47 mRNA, thereby inhibiting CD47 expression. Animal model studies have shown that miR-299-3p expression is suppressed and CD47 is compensatorily upregulated in aortic tissues of Apoe-/- mice fed a high-fat diet (HFD). Restoring miR-299-3p expression through intervention not only significantly inhibits CD47 levels (reducing by approximately 46%) but also promotes the phagocytic clearance of apoptotic foam cells by macrophages within plaques, reducing the necrotic core area, thickening the fibrous cap, and enhancing plaque stability. This provides a potential new target for developing miRNA-targeted gene therapy strategies (83).

## 5 Imaging monitoring of ICI-related atherosclerosis

Imaging techniques are essential tools for monitoring the evolution of atherosclerosis plaques induced by ICIs. Computed tomography (CT), as a routine assessment method, can track the dynamic changes of plaques by comparing enhanced scan data at different time points. Additionally, although the use of 2-[ ^18^F] fluorodeoxyglucose (FDG) positron emission tomography (PET) imaging to evaluate organ inflammation caused by ICI treatment is not yet mature, functional imaging studies have shown that (^18^F -FDG can be taken up by macrophages and foam cells, thus quantifying the inflammatory activity of atherosclerosis after ICI treatment. This method provides an important approach for monitoring the progression of arterial plaques.

A recent retrospective imaging study confirmed the promoting effect of ICIs on the progression of atherosclerosis plaque calcification. The study included patients diagnosed with stage III or IV non-small cell lung cancer (NSCLC) at Wuhan Union Hospital between March 2020 and April 2022, and baseline characteristics were balanced using propensity score matching (PSM). The results showed that during a median follow-up of 23.1 months, the coronary artery calcification (CAC) volume and score significantly increased in the ICI treatment group (84). Coronary artery calcification typically accompanies the development of advanced arteriosclerosis and is a highly specific marker of coronary atherosclerosis.

However, this study primarily focused on the impact of ICI treatment on the degree of coronary artery plaque calcification, while the research by Drobni et al. further revealed the correlation between ICI treatment and the progression of non-calcified plaque volume in the aorta. Notably, the progression rate of non-calcified plaques under ICI treatment was higher than that of calcified plaques. Calabretta et al. reported concordant findings in their cohort study utilizing 18F-FDG PET-CT imaging of 20 melanoma patients receiving ICI therapy (8, 85, 86). Non-calcified plaques are richer in lipids and macrophages compared to calcified plaques, making them more unstable and associated with a higher risk of acute cardiovascular events. It is currently unclear whether the progression of non-calcified plaques in the aorta has similar clinical significance as plaques in the coronary arteries. Future research is needed to further evaluate the impact of ICI treatment on non-calcified plaques in the coronary arteries.

In the latest study by Calabretta et al., 47 lung cancer patients underwent 2- [ ^18^F] FDG PET/CT scans before and after ICI treatment were divided into "pre-existing inflammation" and "no pre-existing inflammation" groups to further analyze the impact of ICI treatment on different plaque subtypes. The results showed that in plaques with "no pre-existing inflammation," arterial ^18^F -FDG uptake significantly increased, while in plaques with "pre-existing inflammation," showed no significant change (13), indicating that ICI may induce vascular inflammation in patients who lack pre-existing arterial inflammation. Vessels without inflammation, due to the immune cells not being depleted, exhibit a more intense immune activation response to ICI. In contrast, vessels with existing inflammation are in a state of chronic low-grade activation, limiting their response to ICI activation. This finding suggests that, rather than simply exacerbating the progression of existing plaques, ICI may accelerate the process of atherosclerosis more by promoting the formation of new plaques.

In summary, CT and PET-CT imaging in patients receiving ICI therapy enable longitudinal assessment of atherosclerotic progression. Furthermore, functional imaging modalities may possess the potential to detect *de novo* plaque formation in patients without baseline vascular inflammation, warranting further validation through dedicated imaging cohort studies.

## 6 Treatment and management strategies for high-risk patients with ICI-related atherosclerosis

### 6.1 Risk factors

Enhancing awareness of risk factors for ICI-related cardiovascular diseases is the first step in preventing ICI-related arteriosclerosis. Retrospective studies have confirmed that cardiovascular risk factors such as BMI, smoking, hypertension, hypercholesterolemia, and diabetes are associated with ICI-related cardiotoxicity (3, 5, 87, 88). However, whether these factors are independent risk factors for ICI-related atherosclerosis remains to be further confirmed. Currently, there is no effective method to identify high-risk patients for immune checkpoint inhibitor-related atherosclerosis. Biomarkers for detecting ICI-related atherosclerosis are a potential effective identification method, but due to the confounding effects of tumor-related pro-inflammatory mediators (such as IL-6, TNF-*α* ), it may be difficult to find these markers. In a latest preclinical mouse model, Vincenzo et al. demonstrated that short-term use of PD-1 and CTLA-4 blockers activated the expression of inflammation pathway-related proteins (NLRP3 inflammasome, MyD88) in the mouse model, and through the NLRP3-MyD88 pathway, activated the downstream pro-inflammatory factor SDF-1, while inducing the release of myocardial injury-related molecules DAMPs (fibronectin-EDA, S100/calgranulin, galectin-3), triggering intense vascular inflammation, suggesting the existence of specific biomarkers for ICIs-related atherosclerosis and new potential intervention targets, which await further experimental exploration (89).

**Figure 3:**
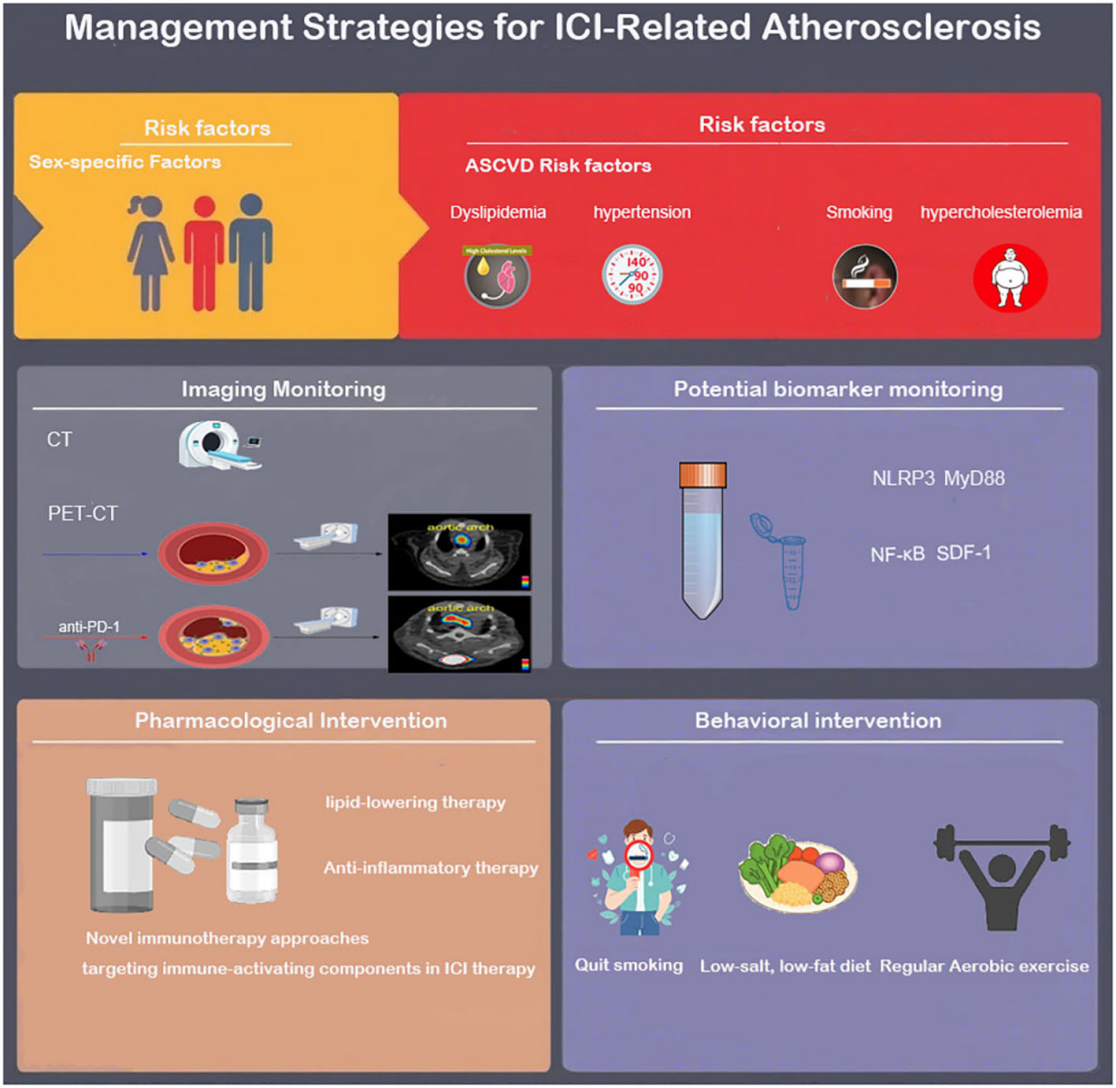
Risk factors, identification and monitoring, treatment, and management strategies for ICI-related atherosclerosis.

It is noteworthy that the impact of ICI on atherosclerosis may be gender-specific, and paying attention to gender differences in ICI-related atherosclerosis can help formulate effective individualized cardioprotective strategies. However, it is still unclear whether the risk of ICI-related atherosclerosis events is higher in men or women. In a retrospective study on ICI-related atherosclerotic cardiovascular events, Bar et al. (5) found that compared to women, ICI treatment increased the odds of AVEs in men by 2.43 times (95% CI 1.04-5.68), although the study had limitations such as a small sample size (n=31) and insufficient representation of female samples. In another retrospective study by Bingxin Gong et al., ICI treatment led to a relatively higher risk of cardiovascular events in women (female HR 12.6 compared to male HR 2.4, P = 0.050) (84). It is worth noting that estrogen may have a more complex immune regulatory mechanism compared to androgen. The interaction between estrogen and estrogen receptors can upregulate the expression of PD-1 and PD-L1, affecting the immunosuppressive function of T cells. Experiments by Magdalena et al. confirmed that in WT mice, E2 (estradiol) treatment upregulated PD-1 expression and enhanced the inhibitory function of Tregs. In PD-1 KO mice, E2 could partially restore Treg function (about 40%) but not to the level of WT mice, indicating that E2 can upregulate the immunosuppressive function of Treg cells through a PD-1-dependent pathway (90). In tumor treatment with PD-1 antibodies, this regulatory pathway may be affected by PD-1 inhibition, thereby impacting the anti-inflammatory role of Treg cells in atherosclerosis. This still requires further experimental confirmation. Currently, there is a knowledge gap in understanding the gender-specific effects of ICI treatment on atherosclerosis (**Figure 3**).

### 6.2 Drug intervention therapy

#### 6.2.1 Lipid-lowering therapy

Statins (hydroxymethylglutaryl-CoA (HMG-CoA) reductase inhibitors) have been proven to achieve the efficacy of stabilizing plaques and reversing endothelial dysfunction by reducing low-density lipoprotein cholesterol (LDL-C) and inhibiting inflammatory responses. Observational studies have demonstrated that the pro-atherogenic effects of immune checkpoint inhibitors (ICIs) may be modulated by statin therapy. Patients receiving statins exhibit a significantly lower annualized progression rate of total atherosclerotic plaque volume compared to non-users (5.2% vs. 8.3%, P = 0.04). Statins are also potentially linked to phagocytosis (91).Both *in vivo* and *in vitro* experiments have demonstrated that statins enhance efferocytosis by inhibiting the nuclear translocation of NFκB1 p50 and downregulating the expression of the critical "don't-eat-me" signaling molecule CD47. This mechanism synergistically enhances the therapeutic efficacy of CD47-SIRPα blockade in anti-atherosclerotic treatment. However, the safety profile of statin therapy in ICI-treated patients without pre-existing cardiovascular disease (CVD) remains undetermined, as statins are associated with a significant risk of muscle injury (85, 92), which may limit their use in patients receiving ICI therapy. Recent studies have confirmed that cancer patients with ICIs-related ASCVD can benefit from non-statin lipid-lowering drugs such as PCSK9 (proprotein convertase subtilisin/kexin type 9) inhibitor therapy. PCSK9 inhibitors (such as evolocumab, alirocumab, etc.) can reduce LDL-C levels by 50%-60% in AVSCD patients and significantly reduce the risk of cardiovascular events. Meanwhile, PCSK9 inhibitors can restore MHC-I expression, enhance cytotoxic T lymphocyte infiltration, and produce synergistic effects with PD-1/PD-L1 inhibitors. In a colon cancer model, the combination therapy of PCSK9 antibody and PD-1 inhibitor increased the proportion of CD8+ T cells in the tumor by 2 times while reducing the number of regulatory T cells (Treg), significantly inhibiting tumor growth. This suggests that PCSK9-targeted therapy has dual therapeutic potential for anti-atherosclerosis and anti-tumor effects (93).

In the context of ICI medication strategies under lipid-lowering therapy, a study showed that in a lipid-lowering environment, the combination of PD-1 and CTLA-4 had better prognostic effects than monotherapy. In the study, Ldlr-/- mice (n=126) were fed a Western diet for 17 weeks (baseline state) and then switched to a standard diet for 4 weeks (plaque progression cessation state), while receiving standard-of-care lipid-lowering therapy. Histology and single-cell RNA sequencing showed that although the degree of cholesterol reduction was comparable to the isotype control group, inhibition of PD-1 or CTLA-4 alone aggravated atherosclerosis and increased the infiltration of T cells and macrophages in plaques, while the combined inhibition group of PD-1 and CTLA4 did not show significant pro-atherosclerotic effects. Moreover, the combined treatment group had thicker plaque fibrous caps and higher collagen content, without significant increase in macrophages and T cells infiltration. This finding provides a new research direction for ICI medication strategies in ASCVD patients undergoing lipid-lowering therapy (94).

#### 6.2.2 Anti-inflammatory therapy

Corticosteroids are commonly used to treat immune-related adverse events (irAEs) in the heart. In a retrospective study conducted by Drobni et al. on patients receiving ICI treatment, patients treated with corticosteroids had a lower annual plaque progression rate compared to those not treated with corticosteroids (3.5% vs 6.9%, P<0.04)). However, considering the adverse effects and immunosuppressive effects of such drugs, the use of corticosteroids in ICI-treated patients is difficult to be used as a routine preventive measure (**Figure 3**).

In recent years, multiple experiments have shown that colchicine has therapeutic effects on atherosclerosis. Colchicine reduces the release of pro-inflammatory cytokines such as IL-1β and IL-18 by inhibiting the activation of the NLRP3 inflammasome, which is highly active in AS plaques, thereby slowing plaque progression (95, 96). The FDA recently approved colchicine anti-inflammatory therapy to reduce the risk of atherosclerotic events (AVEs) such as myocardial infarction (MI) and stroke in adult patients with confirmed atherosclerotic disease or multiple cardiovascular risk factors (97). Notably, animal experiments have confirmed that PD-1 and CTLA-4 blockers activate the NLRP3-MyD88 pathway in mouse models, leading to an increase in pro-inflammatory factors IL-1β, IL-6, and SDF-1, inducing severe vascular inflammation (89). There have been case reports of colchicine successfully treating ICI-related pericarditis and myocarditis. Whether colchicine has the potential to reduce ICI-related atherosclerosis requires further experimental investigation.

#### 6.2.3 Novel immunotherapy approaches targeting immune-activating components in ICI therapy

##### 6.2.3.1 Abatacept and its mutants

Abatacept is a soluble CTLA-4 fusion protein (CTLA-4-Ig), a CTLA-4 analog composed of the extracellular domain of wild-type CTLA-4 and the Fc portion of human IgG1. It can inhibit overactivated immune responses by blocking T cell co-stimulatory signals (CD28-CD80/86 pathway). Recent studies have shown that Abatacept exhibits potential therapeutic effects in atherosclerosis models. Research by Ewing et al. found that in hypercholesterolemic Apoe-/-Leiden mice fed a Western diet, Abatacept could inhibit the activation of CD4+ and CD8+ T cells, significantly reduce the progression of atherosclerosis, and induce a clinically favorable stable plaque phenotype (46). However, as a CTLA-4 analog, Abatacept carries the risk of neutralizing anti-CTLA-4 monoclonal antibodies, thereby reducing the anti-cancer efficacy of anti-CTLA-4. Current research has designed CTLA-4-Ig mutants, such as Belatacept and M17-2, through site-directed mutagenesis, which maintain high affinity for CD80/86 while reducing binding to anti-CTLA-4 antibodies. In mouse models, both significantly reduced T cell infiltration in heart tissue and inhibited CD4+T cell activation as well as the secretion of IFN - γ and TNF-*α*. In MC38, B16, and EG7 tumor models, Belatacept and M17–2 did not affect CTLA-4 or PD-1 antibody-induced tumor rejection. Abatacept mutants (Belatacept and M17) also show potential in treating ICI-induced atherosclerosis, especially by preserving the anti-cancer efficacy of anti-CTLA-4 antibodies. This represents a promising strategy combining ICIs with cardioprotective agents (98, 99).

##### 6.2.3.2 CD8 antibody and IFN-I*γ* antibody therapy

Research by Lisa Detering et al. confirmed that anti-PD-1 treatment in Apoe-/- mice activates CD8+ T cells to release IFN- γ, inducing CXCL9/CXCL10 chemokines, which recruit CCR2+ monocytes/pro-inflammatory macrophages to plaques, exacerbating plaque inflammation. On the basis of anti-PD1 treatment, the addition of anti-CD8 antibodies (targeting clearance CD8^+^T cells) or anti-IFN- γ antibodies (blocking the IFN-γ signaling pathway), compared to control mice receiving only anti-PD-1 treatment, significantly reduced the CCR2 tracer inflammatory signal at plaque lesions under PET-CT, while immunofluorescence staining showed a reduction in CCR2^+^ monocytes/macrophages and CD65^+^ macrophages within the plaques, suggesting a decrease in inflammatory cell infiltration. This indicates a regression in the development of atherosclerotic lesions, suggesting a potential pathway for treating ICI-related atherosclerosis through anti-CD8 or anti-IFN-*γ* therapy.

### 6.3 Behavioral management

In terms of behavioral guidance for patients receiving ICI treatment, patients should be actively encouraged to quit smoking, adopt a low-salt and low-fat diet, and recognize the importance of regular physical activity.

Current research has found that aerobic exercise can protect against atherosclerosis by inhibiting ICI-activated endothelial-mesenchymal transition (EndMT). Vascular endothelial cells undergoing EndMT lose their original tight junction barrier function and acquire characteristics such as high migratory ability and extracellular matrix secretion. ICI treatment increases reactive oxygen species (ROS) levels, triggering oxidative stress, driving EndMT in vascular endothelium, disrupting endothelial barrier function, and secreting pro-inflammatory factors (such as IL-6, TNF-α) and matrix metalloproteinases (MMPs), accelerating plaque progression and instability. The experiment by J. A. Lara Vargas et al. divided C57BL/6 mice carrying melanoma into four groups (IgG control, exercise alone, anti-PD-1 treatment, anti-PD-1 combined with exercise) and monitored their aortic EndMT indicators (100). The results showed that the expression of the EndMT marker vimentin in the arterial protective laminar flow region was significantly increased in the anti-PD-1 monotherapy group (3.8% vs. 0.6% in the IgG group, p=0.03), while this indicator was significantly reduced after combined exercise intervention (0.8% vs. 3.8%, p=0.04), suggesting that exercise can exert anti-atherosclerotic effects by inhibiting EndMT. Further research is needed to clarify the specific regulatory mechanisms of exercise on ICI-induced EndMT and its long-term anti-atherosclerotic effects, in order to develop reasonable exercise strategies for high ASCVD risk patients undergoing ICI treatment (**Figure 3**).

## 7 Conclusion and future expectations

The clinical application of immune checkpoint inhibitors (ICIs) marks an innovative breakthrough in the field of cancer treatment, with ICI therapy now serving as a core therapeutic strategy for various advanced solid tumors. Its efficacy spans diverse patient populations, significantly prolonging progression-free survival (PFS) and overall survival (OS), conclusions that have been confirmed by multiple large-scale clinical studies. However, current research suggests that ICI therapy carries risks of promoting arterial plaque progression, increasing plaque instability, and elevating the incidence of AVEs. This necessitates a balance between tumor eradication and pro-atherogenic effects during ICI treatment, aiming to maximize antitumor efficacy while minimizing atherogenic promotion. Achieving this goal requires deeper understanding of the molecular mechanisms underlying ICI-associated atherosclerosis, particularly the interplay between immune activation, dysregulated inflammatory pathways, and plaque stability. Current research priorities include distinguishing the relative contributions of T-cell reactivation versus *de novo* T-cell recruitment in plaque microenvironment during ICI therapy, and investigating the differential atherogenic mechanisms of CD47, TIGIT, and TIM-3 checkpoint inhibitors compared to established PD-1/CTLA-4 blockade. The potential impact of novel checkpoint targets like CD300Id on atherosclerosis requires experimental validation.

In clinical practice, there is an urgent need to identify predictive biomarkers with diagnostic validity to guide early detection and targeted therapeutic development. Researchers should concurrently advance cutting-edge coronary functional imaging methodologies by developing novel biomarkers superior to ^18^F-FDG for atherosclerosis monitoring. These include molecularly targeted agents such as ^64^Cu-DOTA-ECL1i—a radiotracer specifically binding CCR2 (C-C chemokine receptor type 2) overexpressed on pro-inflammatory monocytes/macrophages and immune checkpoint-directed probes exemplified by zirconium-89-labeled anti-CD40 monoclonal antibodies and indium-111-conjugated CTLA-4 fusion proteins. Collectively, these emerging imaging biomarkers have demonstrated significant potential in clinical trials for identifying and tracking atherosclerotic progression in patients receiving ICIs therapy (12, 101–104). Multidisciplinary collaboration (involving oncology, cardiology, radiology, and pharmacology experts) should optimize risk management through baseline cardiovascular risk stratification for ICI regimen selection, primary prevention with statins or PCSK9 inhibitors, and exploration of novel immunotherapies targeting ICI-related immune activation components. Comprehensive lifestyle interventions (e.g., smoking cessation, exercise guidance) for high-risk ASCVD patients receiving ICIs are crucial to ensure that survival benefits are not compromised by increased cardiovascular morbidity/mortality, ultimately achieving dual benefits in oncological efficacy and cardiovascular safety.
